# Dislocation Mechanism and Grain Refinement of Surface Modification of NV E690 Cladding Layer Induced by Laser Shock Peening

**DOI:** 10.3390/ma15207254

**Published:** 2022-10-17

**Authors:** Yupeng Cao, Pengfei Zhu, Yongfei Yang, Weidong Shi, Ming Qiu, Heng Wang, Pengpeng Xie

**Affiliations:** 1 School of Mechanical Engineering, Nantong University, Nantong 226019, China; 2 Nantong COSCO Shipping Engineering Co., Ltd., Nantong 226001, China

**Keywords:** laser shock peening, high-strength steel, microstructure, grain refinement

## Abstract

To investigate the relationship between the dislocation configuration and the grain refinement in the NV E690 cladding layer caused by laser shock peening, NV E690 high-strength steel powder was used to repair prefabricated pits in samples of 690 high-strength steel by laser cladding, where the laser shock peening of the cladding layer was performed by laser shock at different power densities. X-ray diffraction, scanning electron microscopy, and transmission electron microscopy were used to observe the microstructures of these samples before and after the laser shock process. The results showed that the metallurgical bonding between the cladding layer and the substrate after laser cladding repair was good. When the laser power density was 4.77 GW/cm^2^, multiple edge dislocations, dislocation dipoles, and extended dislocations were distributed over the cladding layer. When the laser power density was 7.96 GW/cm^2^, a geometrically necessary dislocation divided the large original grain into two subgrains with different orientations. When the laser power density was 11.15 GW/cm^2^, geometric dislocations divided the entire large grain into fine grains. The grain refinement model of the NV E690 cladding layer, when treated by laser shock peening, can describe the grain refinement process induced by the dislocation movement of this cladding layer.

## 1. Introduction

The pile legs of offshore engineering platforms have been used in high-pressure, heavy-load, and saline-alkali environments for long periods. The occurrence of pitting corrosion, gluing, and wear on the surface of the lifting rack for these legs will lead to the failure of the lifting rack, which will then affect the overall safety of the offshore engineering platform. Laser cladding repair (LCR) is an advanced technique for the repair and remanufacturing of the surfaces of failed components and offers advantages that include high controllability, high efficiency, and a high degree of automation. LCR is an excellent method for use in the surface repair and remanufacturing of failed workpieces [1,2,3,4]. However, during the repair process, defects, e.g., pores, cracks, and uneven residual stress distributions can form because of the uneven process temperature distribution and unsuitable process parameters [5,6]. As a new method to strengthen the surfaces of metal materials, laser shock peening (LSP) can reduce the number of defects in the laser cladding layer, refine the grain structure in the laser cladding layer, and improve the residual stress distribution through impact strengthening treatment [7,8]. Under the action of a laser-induced shock wave, the dislocation source in the material generates new dislocations continuously, and these new dislocations, which move at high-speed, will cause a change in the original configuration and thus have a significant effect on the macroscopic mechanical properties of the material [9,10,11,12,13]. In recent years, researchers have focused on both the microstructural evolution and the strengthening mechanism of materials before and after LSP, and they have established the strengthening mechanisms for stainless steel, aluminum alloys, and several other materials [14,15,16,17]. However, dislocation configuration has not been used to investigate the specific defect types that occur in the cladding layer after LSP, and its relationship with the grain refinement process must also be established.

In this study, NV E690 high-strength steel powder was used to repair prefabricated pits in specimens made from the commonly used pile leg material 690 high-strength steel, and LSP of the cladding layer surface was then performed. Cross-sectional scanning electron micrographs, surface transmission electron micrographs, and high-resolution transmission electron micrographs of the specimens were acquired before and after LSP using a scanning electron microscope (SEM) and a transmission electron microscope (TEM), and the high-resolution TEM (HRTEM) images were then subjected to inverse fast Fourier transform (IFFT) processing. The interface relationships between the precipitated phase and the substrate, the defect types, and the multiscale relationship between the dislocation configuration distribution and the grain refinement were investigated. A model of the grain refinement of the NV E690 cladding layer produced by LSP was established from a dislocation configuration perspective that provides a theoretical basis for the repair and process optimization of offshore equipment components made from 690 high-strength steel.

## 2. Experiments

### 2.1. Material and Specimens

**Figure 1:**
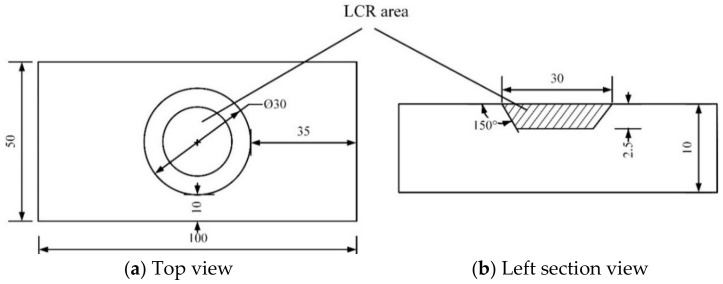
Schematics of the top and left-hand side views of the flat specimens.

The substrate material was 690 high-strength steel and the specimen dimensions were 100 mm × 50 mm × 10 mm. A disc punch and 80~1500 mesh water sandpaper were used to grind the sample pasted on the manual grinding plate to 0.06 mm along the back, followed by the use of a high-precision pit instrument to grind the sample along the back to 15 μm. Finally, the pitted sample was ion-thinned until a large and uniform thin area appeared. Specimen 1 was treated by laser cladding alone, and specimens 2–4 were treated by LSP after LCR. Based on consideration of the common pitting defects that occur on the surfaces of pile leg lifting racks, typical truncated cone-shaped pits with a maximum diameter of 30 mm, depth of 2.5 mm, and an angle of 150° between the frustum-shaped pit and the upper surface were prefabricated at the center of the specimens. The dimensions of the flat sample are illustrated in Figure 1. The cladding repair powder was NV E690 high-strength steel powder with particle sizes ranging from 45–105-μm and a purity of 99.9%. The chemical compositions of both the substrate and the powder are given in Table 1.

### 2.2. Laser Cladding and LSP Experiments

The LCR experiments were performed using the LCR test bench at Southeast University. The main components of this laser cladding bench were a laser system (TRUMPF Laser Trudiode 3006; maximum output power: 3000 W; wavelength: 1030 nm), a coaxial nozzle (YC52), a double-barreled powder feeder, and a four-axis numerical control machine tool (AFS-1280). Coaxial nitrogen was used as a protective gas during the LCR process. The protective gas flow rate was 6 L/min, the pressure intensity of synchronous coaxial powder feeding was 0.6 MPa, and the scanning path was a spiral from the outer edge of the prefabricated pit to the center of the circle. Based on previous results obtained by our research group, the optimized process parameters are given in Table 2.

**Figure 2:**
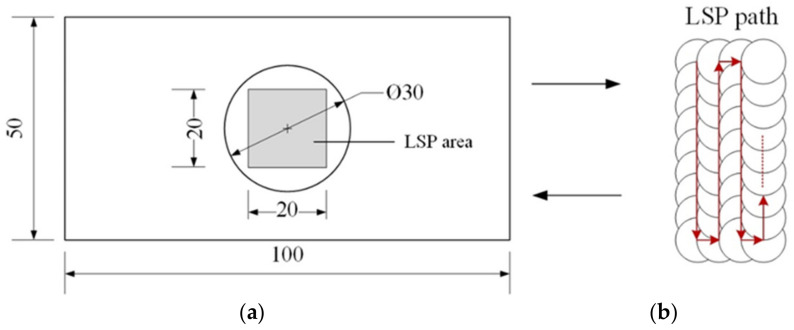
(**a**) LSP treatment area, and (**b**) LSP scanning path.

The surfaces of specimens repaired by laser cladding were polished using a DPF-2 electrolytic polisher, cleaned using absolute ethyl alcohol, and naturally air-dried, and specimens 2–4 were then submitted to LSP treatment after polishing. The LSP experiments were performed using a Q-switched Nd:YAG laser system (SGR, Beamtech, Beijing, China) with an operating wavelength of 1064 nm and a pulse width of 20 ns. In this work, the laser spot diameter was 2 mm and the overlap ratio was 50%. The laser pulse energies were set at 3, 5, and 7 J, and the corresponding laser peak power densities were 4.77, 7.96, and 11.15 GW/cm^2^, respectively. The area treated by LSP had dimensions of 20 mm × 20 mm. Aluminum foil with a thickness of approximately 0.1 mm was used as an absorption coating to protect the specimens from thermal effects, and a deionized water film with a thickness of approximately 2 mm was used as a transparent confining layer. The LSP treatment of the surfaces of specimens 2–4 was performed once. The overlapping scheme for the light spots and the affected area is shown in Figure 2.

### 2.3. Microstructural Observation

**Figure 3:**
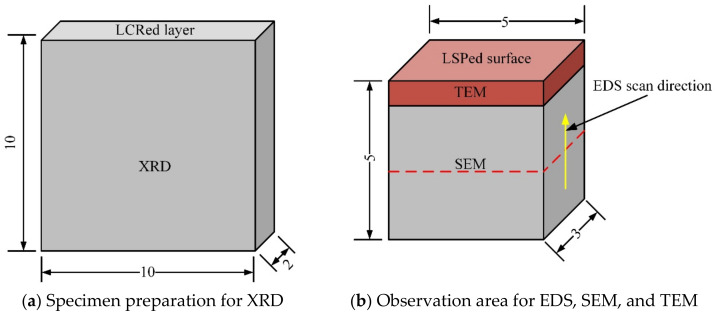
Schematic diagrams of the samples and observation areas used for the various test methods.

Specimen 1 was repaired by laser cladding and was then cut to a sample size of 10 mm × 10 mm × 2 mm (Figure 3a), and phase analysis of the laser cladding layer was then performed via X-ray diffraction (XRD; Ultima IV, Rigaku, Tokyo, Japan). XRD continuous scanning was used during the XRD process. 2θ ranged from 5° to 90°, the scanning speed was 4°/min, and the interval between the data points was 0.02°.

Using the upper surface of the laser cladding layer as a reference plane, the specimens that were processed by LCR alone and by combined LCR and LSP treatment were cut into 5 mm × 3 mm × 5 mm pieces along the direction perpendicular to the laser scanning path in the LCR. The microstructural morphology of the specimens was observed in the cross-section direction using an SEM (Quanta 650F, FEI, Hillsboro, OR, USA), and the microstructural morphology of the specimen’s surface layer was observed via a TEM (Tecnai G2 F20, FEI, Hillsboro, OR, USA). The distribution of the elements was analyzed by scanning electron microscopy with energy dispersive spectroscopy (EDS). A schematic diagram of the specimen that shows the observation direction is shown in Figure 3b.

## 3. Results and Analysis

### 3.1. Analysis of the Microstructure of the Laser Cladding Layer

#### 3.1.1. Phase Analysis by XRD

**Figure 4:**
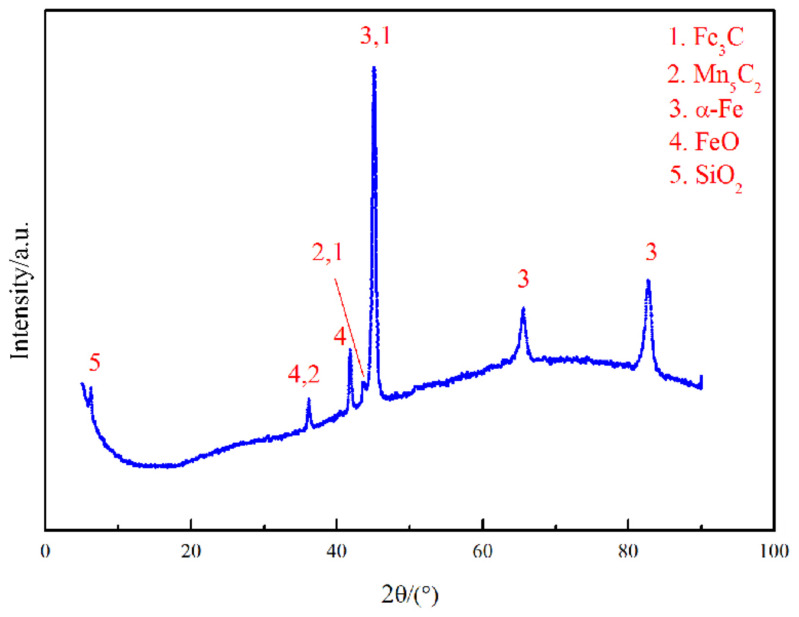
XRD pattern of the cladding layer of specimen 1.

By performing search matching of the XRD pattern, XRD phase analysis results were obtained for the laser cladding layer (Figure 4). The laser cladding layer is principally composed of an α-Fe substrate and other precipitates that mainly consist of FeO, SiO_2_, Mn_5_C_2_, and Fe_3_C. It can be reasonably inferred that as an oxyphilic element, the Si in the molten pool easily combines with O to form SiO_2_. At the same time, a small amount of Fe is combined with O to form FeO, Mn is combined with C to form Mn_5_C_2_, and residual C is combined with Fe to form Fe_3_C. 

#### 3.1.2. SEM Analysis of LCR

**Figure 5:**
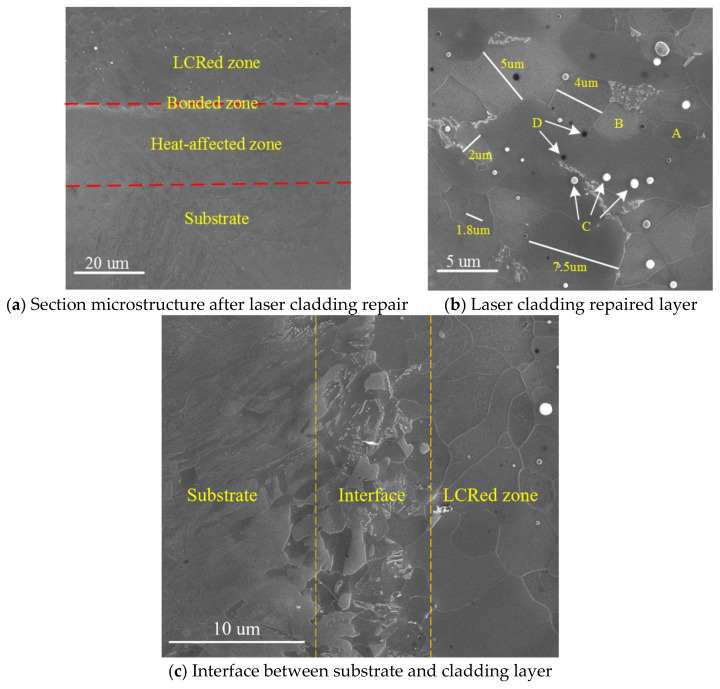
SEM morphology for specimen 1.

SEM images of a section of specimen 1 are shown in Figure 5. According to the different microstructures shown in Figure 5a, there were four distinct areas in the specimen (from top to bottom): the laser cladding layer, the bonding zone, the heat-affected zone, and the substrate. The cladding layer microstructure was that of an equiaxed grain, and no cracks, pores, or other defects were observed.

The microstructural grains in the cladding layer were equiaxed grains that had obvious grain boundaries, along with grain sizes of between 1.8 and 7.5 μm (Figure 5b). The average surface grain size measured using the average intercept method was 4.0 μm. The laser cladding layer microstructure was composed of the gray grain structure A, the light-white grain structure B, the white spherical precipitate C, and the black precipitate D. The elemental contents of each phase were determined by EDS, and the contents of each phase shown in Figure 5b are given in Table 3. According to the EDS results, both gray grain A and light-white grain B mainly contained Fe, the white spherical precipitate C mainly contained C, O, Si, Mn, and Fe, and the atomic number ratio of C to Mn was close to 2:5. The black precipitate D mainly contained C and Fe, and the atomic number ratio of C to Fe was close to 1:3. When combined with phase analysis performed by XRD (Section 3.1.1), the EDS results were used to determine that both the gray grain A and the light-white grain B were composed of ferrite with a BCC structure, and the white spherical precipitate C was composed of a mixture of SiO_2_, FeO, and Mn_5_C_2_, although the mixing ratio of SiO_2_ and FeO was unknown [18,19]. The black precipitate D was composed of spherical Fe_3_C. When the liquid phase changes into the solid phase, the balance between these two phases should be satisfied. The local composition changes when grains grow from the alloy melt. Since the melting point of iron is higher than that of manganese, iron solidifies faster [20]. During solidification in the molten laser cladding pool, the Mn content of the molten pool increased because of some grains that were first nucleated and then solidified, and the later-solidified grains were enriched with a small amount of Mn. Figure 5c is a magnified view of the interface between the cladding layer and the substrate. The cladding layer and the substrate form a good metallurgical bond, and no obvious defects are observed.

### 3.2. TEM Analysis

#### 3.2.1. TEM Image of the Cladding Layer

**Figure 6:**
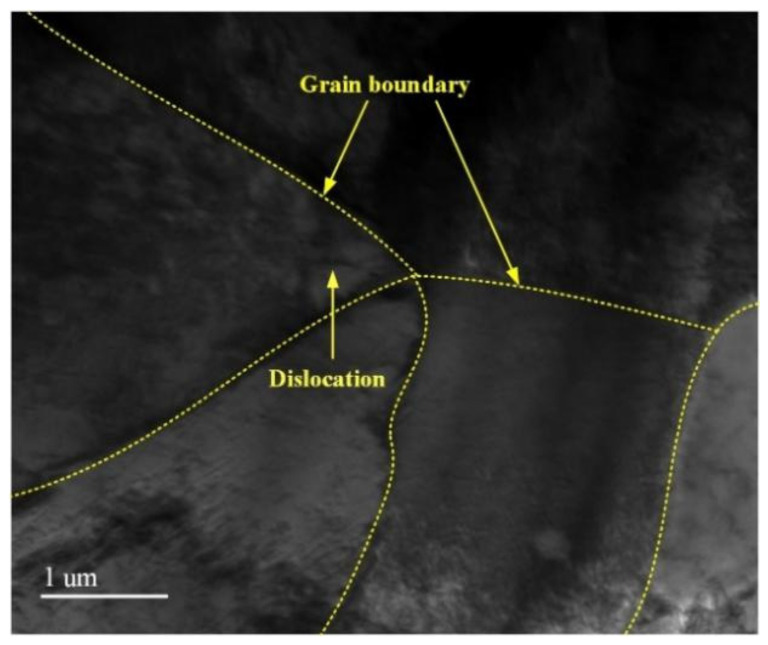
Typical TEM image without LSP.

A TEM image of a typical cladding layer without LSP is shown in Figure 6. Combined with the SEM image of the cladding layer in Figure 5b, the cladding layer had an equiaxed crystal microstructure, the microstructure in the grains was uniform, the grains were coarse, and a few dislocation lines were randomly distributed among the original grains. During solidification in the laser cladding, the growing grains met. When the molten pool cooled, under the comprehensive action of the temperature gradient, structural fluctuations, and structural changes, local internal stress then formed in the cladding layer, which led to the formation of a small number of dislocation lines in the NV E690 cladding layer [21].

#### 3.2.2. TEM Image after LSP

**Figure 7:**
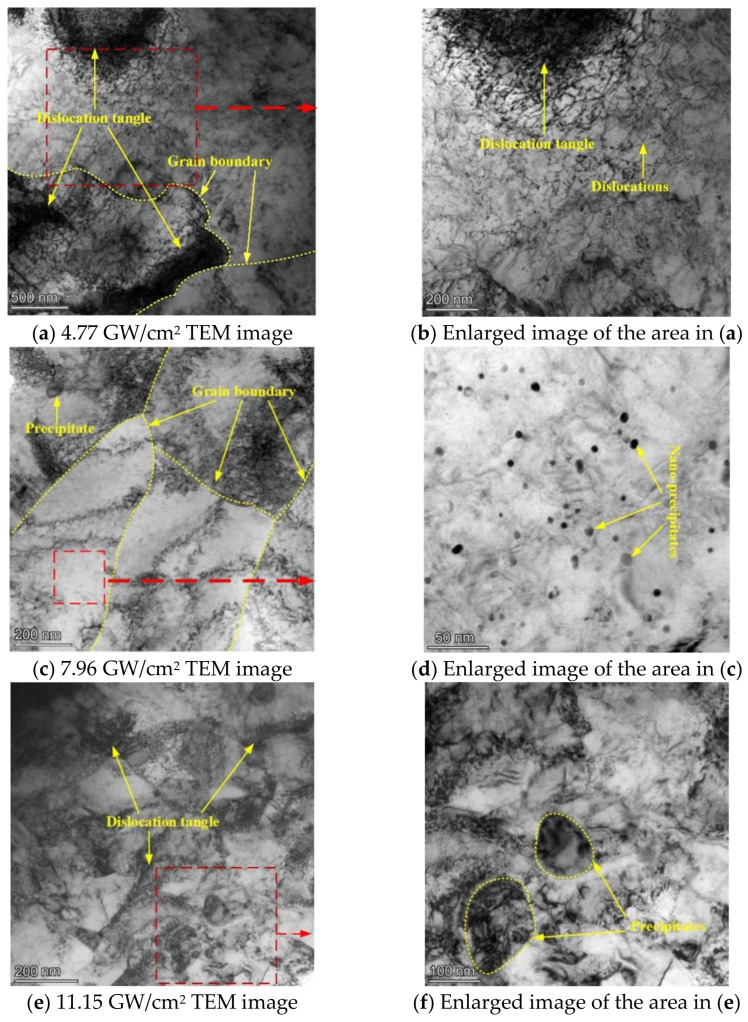
TEM images acquired after LSP at different power densities.

TEM images of cladding layers after LSP treatments at different power densities are shown in Figure 7. A TEM image of the NV E690 cladding layer after LSP at a power density of 4.77 GW/cm^2^ is shown in Figure 7a, and an enlarged image of the area indicated by the dashed square in Figure 7a is shown in Figure 7b. The grains in the cladding layer in this case were coarse, and dislocation tangles formed near the grain boundaries because of obstruction of the grain boundaries. When compared with the TEM image of the typical cladding layer before LSP (specimen 1, as shown in Figure 6), the dislocations multiplied in the grains, and dislocation tangles formed near the grain boundaries. A TEM image of the cladding layer after LSP at a power density of 7.96 GW/cm^2^ is shown in Figure 7c. The grain size was obviously reduced when compared with the grains obtained following LSP at the power density of 4.77 GW/cm^2^ (Figure 7a), and the dislocations were multiplied both in the vicinity of the grain boundary and inside the grain. An enlarged image of the area indicated by the dashed square in Figure 7c is shown in Figure 7d. The image in Figure 7d shows that many nanoscale precipitates were present in the cladding surface of the NV E690 cladding layer, which hindered dislocation movement and thus strengthened the material. A TEM image of the cladding layer after LSP at a power density of 11.15 GW/cm^2^ is shown in Figure 7e, and an enlarged image of the area indicated by the dashed square in Figure 7e is shown in Figure 7f. Figure 7e shows that the grain size decreased further at the higher power density and that nanograins also appeared in some areas. The dislocations in the grains multiplied further, and dislocations in different directions were intertwined to form complex dislocation configurations. When compared with the original cladding layer structure, only small numbers of the precipitated phases were distributed (Figure 7f). The phenomena described above indicate that after LSP at the different power densities, dislocations, precipitated phases, and fine-grain strengthening processes occurred in the E690 high-strength steel cladding layer, and the surface modification of the E690 high-strength steel cladding layer was thus realized.

### 3.3. Investigation of the Surface Dislocation Configuration of the NV E690 Cladding Layer Formed by LSP

Numerous dislocations were produced in the NV E690 cladding layer by LSP. The dislocations in the crystal can only terminate at the grain boundary, outcrop on the material surface, or connect with other dislocations [22]. Based on a combination of the geometric and energy conditions, when LSP was performed, the high-speed moving dislocations inside the material would undergo dislocation reactions, e.g., decomposition and merging. When two dislocations that had opposite Burgers vectors approached each other, they would then “annihilate” as a result of mutual attraction [22]. Because of the effect of the lap ratio, some regions were subjected to multiple laser shock loading, and interactions between the original dislocation and the new dislocation would also occur. The substrate structure and the precipitated particles were relatively soft, and most of the shock wave energy was unloaded via deformation, which resulted in aggregation of the grain defects in the substrate structure, producing a variety of configurations.

**Figure 8:**
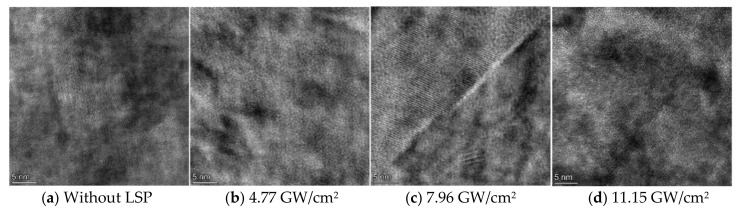
HRTEM images of the laser NV E690 cladding layer without and after LSP at various laser power densities.

**Figure 9:**
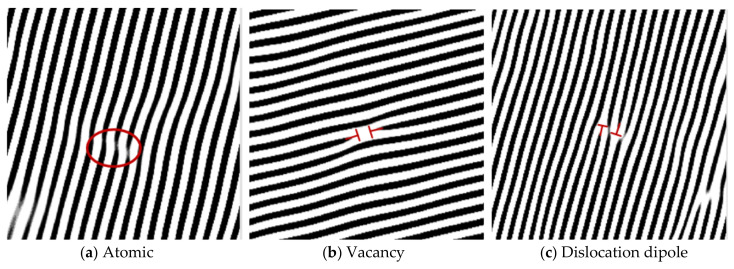

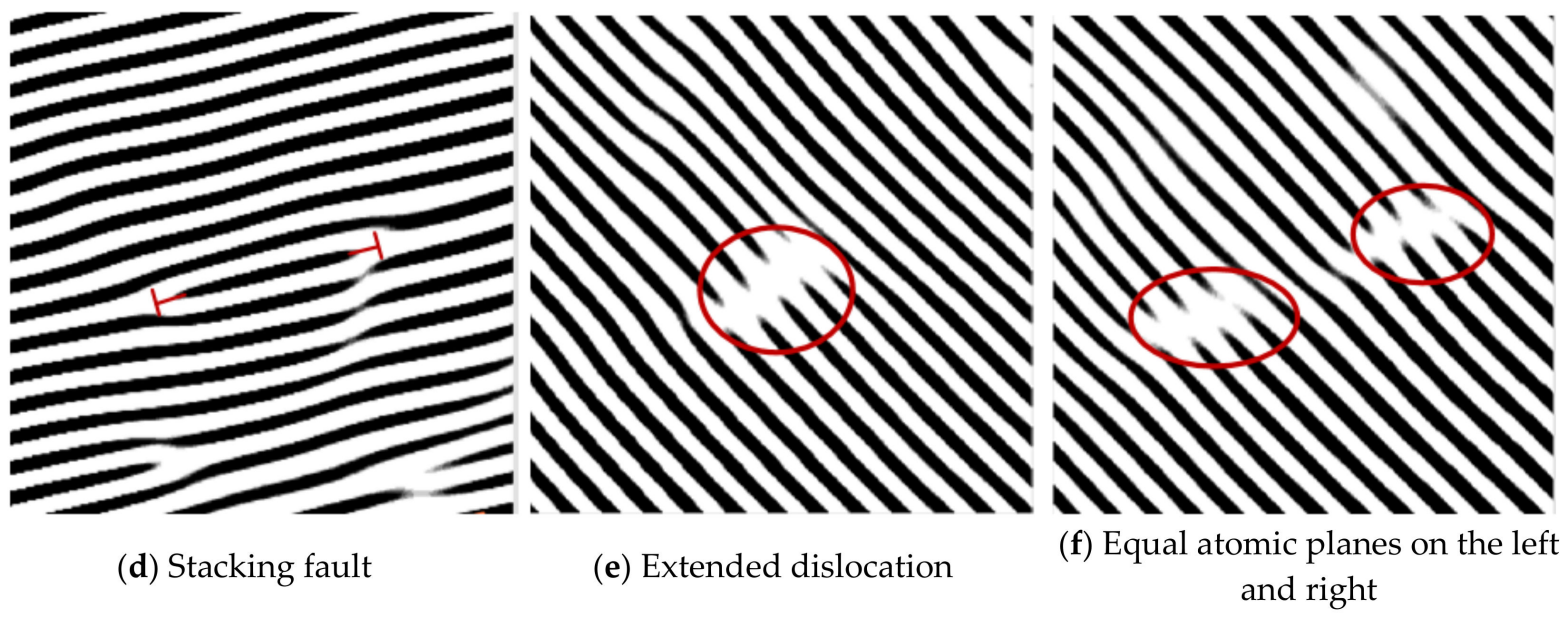
Different types of dislocation within the NV E690 cladding layer after LSP.

HRTEM images of the substrate structures of NV E690 cladding layers without LSP and after LSP at different laser power densities are shown in Figure 8. The contrast of the substrate area changed obviously with the LSP treatment, thus indicating that many grain defects were present in the substrate. Through the IFFT treatment of the images in Figure 8a–d), characteristic images of the different defects in the substrate structure were obtained and are shown in Figure 9. As a result of atomic shearing (Figure 9a), the shear displacements of the atoms caused some lattice distortion during LSP. A vacancy, which is in an unstable state, is shown in Figure 9b. Vacancies can not only promote dislocation formation but also combine with other atoms to produce annihilation during LSP. A dislocation dipole, which was a commonly observed defect in the cladding layer, is shown in Figure 9c. Dislocation dipoles have Burgers vectors that are parallel but are oriented in opposite directions, and when they are close to each other, these dipoles are easily annihilated. A stacking fault is shown in Figure 9d. The NV E690 cladding layer had high stacking fault energy, and there was also a small distribution of these crystal defects in the substrate. A central dislocation band with strip vacancy sheets formed by dislocation extensions in several atomic rows is shown in Figure 9e. A series of special configurations composed of different dislocation groups that are distributed widely within the cladding layer is shown in Figure 9f. Unlike the extended dislocation case, the numbers of atoms on both sides are equal, which not only maintains the balance between the lattice atoms but also causes lattice distortion and thus strengthens the cladding layer material [23].

**Figure 10:**
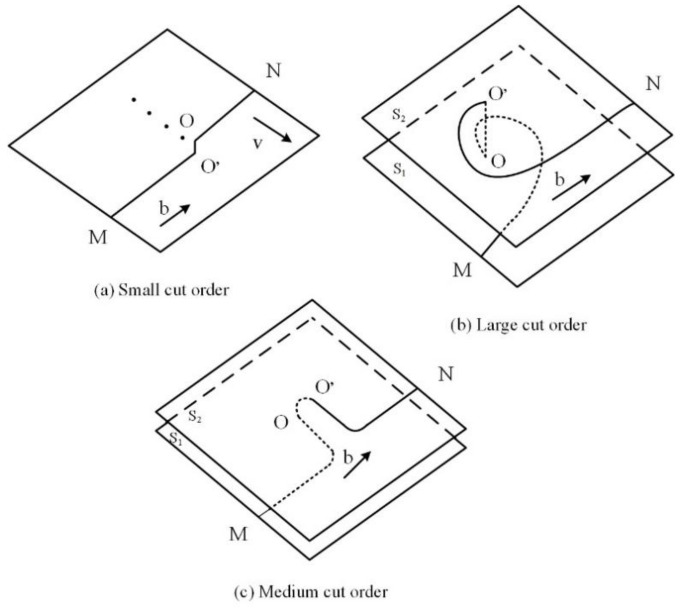
Schematic diagrams of screw-type dislocation movements at different cutting step heights.

Dislocation dipoles are grain defects that commonly occur in metal materials during cold working. The effects of multiple dislocation dipole aggregations on the macroscopic properties of materials cannot be ignored [22]. The formation of these defects is related to the movement of screw dislocations with dislocation jogs, as illustrated in Figure 10. When the height of the dislocation jog OO′ is only on the scale of a few atoms, the screw dislocation MN will then drag the dislocation jog OO′ together under the action of strong external shear stress and will leave a series of point defects on the movement path, as shown in Figure 10a. When the height of the dislocation jog OO′ reaches 20 nm, the dislocation jog is then equivalent to pinning because of its long length, and the dislocation lines of the upper and lower parts of the dislocation jog are located far apart and have little effect on each other. The dislocations at the two ends form a straight line, where the dislocation jog acts as the axis of rotation, and the line rotates on the sliding surface, as shown in Figure 10b. When the dislocation jog height OO′ is within the range of both dislocations, it is then difficult for the dislocation line MN to move forward with the dislocation jog OO′, and the section of the dislocation line affected by the dislocation jog will then bend under the action of the applied stress. Along with the continuous movement of the dislocation, a set of elongated dislocation lines with different signs (also called a dislocation dipole) remains on the slip surface, and the direction of the dislocation line is then perpendicular to that of its Burgers vector, which can be regarded as a formation composed of a cluster of edge dipoles, as shown in Figure 10c. The internal dislocations of the NV E690 cladding layer are multiplied by LSP, and the dislocation dipoles are distributed in pairs. Dislocation dipoles occurring in the same atomic row and several atomic rows of the material are shown in Figure 9c,d, respectively.

**Figure 11:**
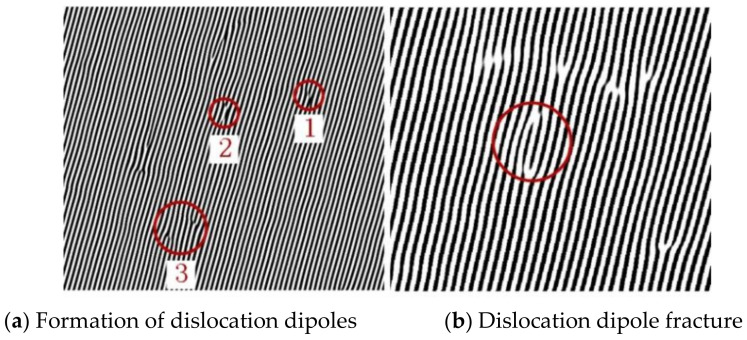
Formation and development of dislocation dipoles in the NV E690 cladding layer after LSP.

A certain number of dislocations will be generated when the crystals of the laser cladding layer are formed. LSP causes the material that is loaded by the laser energy to be far from its thermodynamic equilibrium state, and the internal defects of the grains will change accordingly. The dislocation system is able to achieve a dynamic equilibrium inside the system through a combination of energy dissipation and self-organization [23]. Based on the properties of the dislocations in the grains, the dislocations slip within the crystal, and their types and characteristics also change during the sliding process that occurs during LSP. The formation of dislocation dipoles is illustrated in Figure 11. During the LSP treatment (Figure 11a), the lattice at position 1 is distorted, which results in a complete atomic row becoming a two-edge dislocation. At the same time, this dislocation should not only slip but should also keep the local lattice spacing from changing greatly. Two-edge dislocation slips occur in opposite directions, as shown at position 2. After a certain slip distance, when the distortion stress around the dislocation and the external residual stress reaches a relative balance between them, the movement stops, and a dislocation dipole is formed opposite to the Burgers vector, as shown at position 3. The circle in Figure 11b highlights a dislocation dipole. This dislocation dipole is formed by the dislocation of atoms after shearing and breaking, and higher residual stress will be formed in this area [23].

### 3.4. Surface Grain Refinement of the NV E690 Cladding Layer by LSP

LSP is from the field of cold working technology and relies on the mechanical effect of its detonation wave to improve the surface properties of materials. The internal temperature changes of the materials during the shock process have not been measured. The recrystallization temperature of the NV E690 cladding layer is usually between 0.6 and 0.8 times the melting point of the material, *T*_m_. The environments of the atoms in the different regions will still be different, and the atoms in the different regions cannot realize the diffusion transfer process that occurs during the recrystallization process in a very short time with LSP. In addition, it is difficult to explain the cause of the formation of many fine grains in the original grains based on only the internal solid impurity particles and the heterogeneous nucleation occurring at the grain boundaries. Therefore, many fine grains may be formed on the cladding layer surface under intense laser shock loading by instantaneous self-organization and differentiation of the original coarse grains.

**Figure 12:**
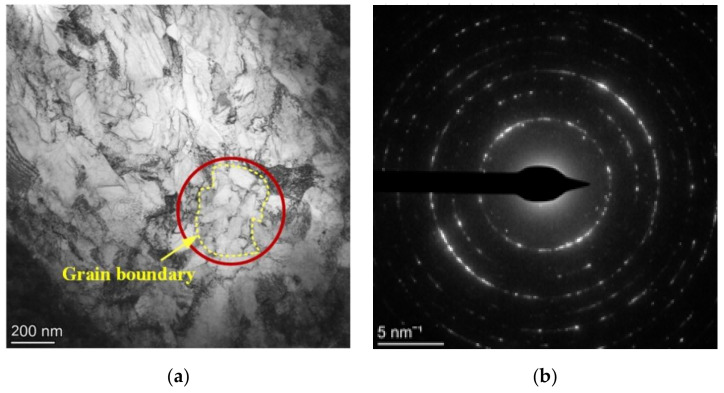
(**a**) TEM morphology and (**b**) selected-area electron diffraction pattern of the cladding layer surface of an E690 high-strength steel sample after LSP.

The grain sizes in the NV E690 cladding layer differed after LSP was performed at different energies (Figure 7). A TEM image and the selected area electron diffraction pattern of a surface after LSP at 11.15 GW/cm^2^ are shown in Figure 12. When the power density was less than 11.15 GW/cm^2^, the grain size of the surface layer of the E690 high-strength steel during intense laser loading of the observation area was mostly more than 150 nm (Figure 7). When the energy reached 11.15 GW/cm^2^, as shown in Figure 12a, the grain refinement was remarkable, and complete large grains were divided continuously by the dislocations to form multiple fine grains, most of which had grain sizes of less than 100 nm. Electron diffraction analysis of the selected region showed that the electron diffraction pattern formed a concentric ring (Figure 12b), which indicated that the grains in this region were nanocrystals with random orientations and uniform distribution.

**Figure 13:**
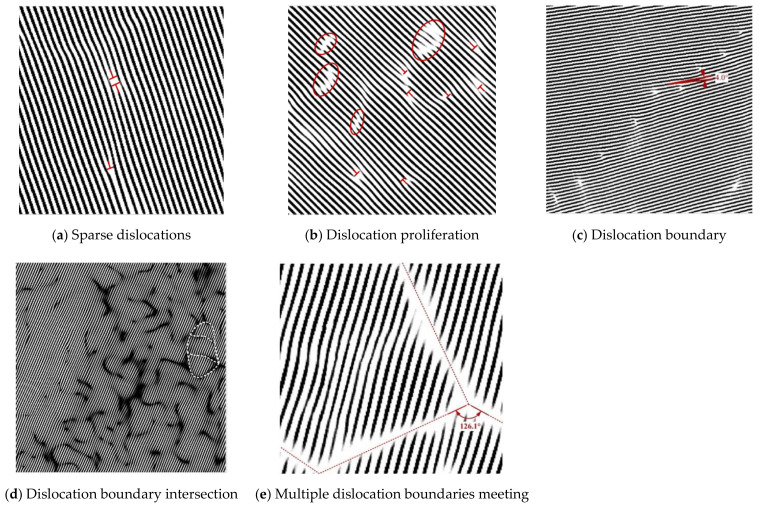
Characteristics of variation of the IFFT-filtered images of the NV E690 cladding layer surface caused by LSP.

Through IFFT processing of the HRTEM images of the cladding layer without LSP and the corresponding images acquired after LSP with different energies, IFFT-filtered images of the cladding layers composed of E690 high-strength steel were obtained. As a result of comparative studies of the dislocation variation characteristics, the specific characteristics are presented in Figure 13, and the grain refinement process that was dominated by dislocations in the E690 cladding layer after LSP was investigated. The dislocations located within the cladding layer before LSP were sparse, with only a few edge dislocations and vacancies being observed (Figure 13a). The dislocation-change characteristics of the IFFT-filtered image of a typical HRTEM image of the cladding layer acquired after LSP at 4.77 GW/cm^2^ are shown in Figure 13b. The dislocation-change characteristics indicate that all defect types proliferated, resulting in the occurrence of numerous strip vacancy components and dislocation dipoles. The dislocation-change characteristics of the IFFT-filtered image of the typical grain HRTEM image of the cladding layer acquired after LSP at 7.96 GW/cm^2^ are shown in Figure 13c. When compared with the cladding layer after LSP at 4.77 GW/cm^2^ (shown in Figure 13b), because of the plastic deformation, and in addition to the interior of the cladding layer producing some randomly distributed dislocations, the dislocations, extended dislocations, and vacancies that occurred in some regions with strong constraint abilities constituted geometric dislocation interfaces. These interfaces divided an original coarse grain into two subgrains with different orientations, depending on the specific constraint conditions and geometric conditions in the crystal. The dislocation-change characteristics of the IFFT-filtered image of the HRTEM image of the nanograin area in the cladding layer indicate that further increases in the grain orientation difference under external loading will lead to gradual changes in the grain-boundary characteristics until a large-angle grain boundary is formed (Figure 13d). When compared with the cladding layer after LSP at 7.96 GW/cm^2^ (Figure 13c), the dislocation interfaces in the different directions expanded and then converged to a single point, thus forming high-angle grain boundaries with each other, and the grains were then refined further. In the nanograin area of the NV E690 cladding layer (Figure 13e), the geometric dislocation interface extended gradually to the entire grain and then divided the whole grain into several small grains. It can be inferred from this behavior that the fine grains in this area were derived from the continuous self-organization differentiation of the larger grains.

**Figure 14:**
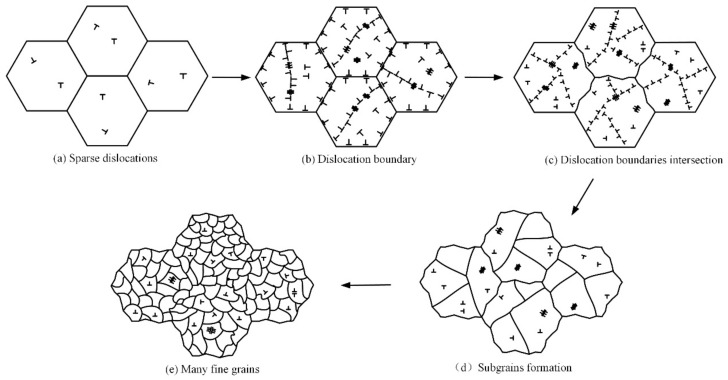
Grain refinement model dominated by the dislocation movement of the E690 high-strength steel cladding layer fabricated by LSP.

Under the conditions described in the experimental scheme design, based on the dislocation movement characteristics in the NV E690 cladding layer and the grain refinement process caused by this layer’s expansion and intersection, and by considering the research results of Lu and Ren on the grain refinement of an LY2 aluminum alloy and a nickel alloy by LSP [24,25], the grain refinement mechanism induced by the dislocation movement of the E690 high-strength steel cladding layer caused by LSP was established (Figure 14). Each untreated E690 high-strength steel cladding layer was an equiaxed crystal, and the dislocations inside the grain were sparse and distributed discretely (Figure 14a). During LSP, atomic shear and dislocation multiplication occurred in the cladding layer, and common defects, e.g., vacancies, edge dislocations, and dislocation dipoles, were formed. The dislocations in the grains were hindered by the grain boundaries and gathered near these grain boundaries to form dislocation tangles. Under the action of the high strain rate induced by the shock wave, the dislocations multiplied further, and the different defects gradually converged to form low-angle grain boundaries. The entire grain was divided into subgrains that had different orientations (Figure 14b). Because of the effects of the ultra-high strain rate induced by the strong shock wave, the grain boundary orientation differences increased further, and the dislocation interfaces in the different directions expanded and met at one point to form a high-angle grain boundary. Some grains were affected multiple times, and the original grain boundaries then began to bend under the stress of the dense dislocations (Figure 14c). Under the action of the ultra-high strain rate, a series of dislocation walls formed and divided the large grains into a series of dislocation cells (Figure 14d). During the expansion of the geometric dislocation interface, multiple small grains gradually formed from the subgrain boundary. The original grain boundary was divided gradually, and the original coarse grains then self-organized and differentiated into fine grains (Figure 14e).

## 4. Conclusions

1. The laser cladding layer was mainly composed of a ferrite base phase, and it had an equiaxed grain microstructure. Hard precipitates, e.g., SiO_2_, FeO, Mn_5_C_2_, and Fe_3_C, were dispersed and distributed, and a good metallurgical bond was observed between the cladding layer and the substrate.
2. Changes in laser power density had a significant effect on the dislocation configuration on the sample surface of the cladding layer under LSP. When the laser power density was 4.77 GW/cm^2^, multiple edge dislocations, dislocation dipoles, and extended dislocations were distributed over the cladding layer after LSP. When the laser power density was 7.96 GW/cm^2^, a geometrical dislocation interface consisting of dislocations, extended dislocations, and vacancies appeared on the surface of the cladding layer, which divided the original large grain into two subgrains with different orientations. When the laser power density was 11.15 GW/cm^2^, the complete large grains were divided into fine grains by the expansion and intersection of geometric dislocation interfaces on the surface of the sample’s cladding layer.
3. During the LSP process of the NV E690 cladding layer, multiple dislocation defects were present in the material, including single-edge dislocations, extended dislocations, and dislocation dipoles. The formation of the dislocation dipoles was closely related to the movement of the screw dislocations with the dislocation jogs. Geometrical dislocation interfaces composed of dislocations, extended dislocations, and vacancies divided the original coarse grains into finer grains until they were divided into fine and uniform nanograins.
4. A grain refinement mechanism dominated by dislocation motion in the NV E690 cladding layer after LSP was established, and this mechanism describes the grain refinement process dominated by the dislocation movement. Surface modification of 690 high-strength steel specimens can be achieved through dislocation strengthening and dislocation-induced refined grain strengthening. However, the constraints and the geometric conditions required for the formation of these geometric dislocation interfaces in 690 high-strength steel by LSP will still have to be investigated further.

## Figures and Tables

**Table 1:** Chemical compositions of the substrate and powder (mass fraction (%)).

Alloy	C	Si	Mn	P	S	Cr	Ni	Mo	V	Fe
Substrate	0.15	0.50	1.52	0.03	0.01	1.50	3.60	0.72	0.06	Bal.
Powder	0.15	0.29	1.35	0.03	0.01	0.16	0.24	0.15	0.06	Bal.

**Table 2:** Process parameters for the LCR process.

Pulse Width (ns)	Laser Power (W)	Diameter of Laser Spot (mm)	Overlap (%)	Powder Feeding Rate (g/min)	Laser Scanning Speed (mm/min)
15	1000	2	62.5	6	700

**Table 3:** EDS analysis results for each area in the cladding layer in specimen 1 (atomic fraction (%)).

Area	C	O	Si	Mn	Fe
A	2.14	0.93	0.20	0.91	95.81
B	1.64	0.57	0.36	1.69	95.73
C	10.09	41.14	8.89	24.81	15.05
D	23.49	0.43	0.19	1.81	74.07

## Data Availability

Not applicable.
